# Biomechanical study of robot-navigated targeted cross-puncture percutaneous vertebroplasty for Kümmell's disease: finite element analysis

**DOI:** 10.3389/fresc.2026.1709907

**Published:** 2026-04-08

**Authors:** Haibin Zhou, Chunjiu Gao, Xiaoyuan Zhang, Bao Chen, Xiao Meng, Yifeng Liao, Xuebin Tang, Hua Li, Yunqing Wang

**Affiliations:** 1 Department of Orthopedics, The Second Affiliated Hospital of Xuzhou Medical University, Xuzhou, China; 2 Graduate School of Xuzhou Medical University, Xuzhou, Jiangsu, China

**Keywords:** finite element analysis, Kümmell's disease, orthopedic navigation robot, percutaneous vertebroplasty, targeted cross-puncture

## Abstract

**Objective:**

The objective of this study is to utilize finite element analysis (FEA) to compare the biomechanical advantages and disadvantages of bone cement distribution patterns during robot-navigated targeted cross-puncture percutaneous vertebroplasty (PVP) for osteoporotic vertebral compression fractures (OVCF) complicated by Kümmell's disease.

**Method:**

A patient-specific finite element model of the T12–L2 segment was developed from CT data to simulate L1 Kümmell's disease with an intravertebral vacuum cleft (IVC). The model simulated two surgical approaches: conventional percutaneous vertebroplasty (CPVP; cement filling was limited to the IVC) and robot-navigated targeted cross-puncture PVP (RPVP; incorporating a subjacent cancellous bone cement anchor). Material properties were assigned, and the lower surface of the L2 vertebral body was fully constrained to define the boundary conditions. Subsequently, six physiological loading conditions (flexion, extension, left/right lateral bending, and left/right axial rotation) were applied under a combined load of 500 N compressive preload and 7.5 N·m moment. Biomechanical outcomes included the von Mises stress distribution in the bone cement, vertebral body, and T12 inferior endplate, as well as the relative displacements.

**Results:**

Compared to CPVP, RPVP demonstrated superior biomechanical stability, evidenced by significantly reduced cement displacement and improved segmental stability in the T12–L2 functional spinal unit. RPVP also lowered peak vertebral stress in the vertebral body of L1, suggesting enhanced load distribution. A trade-off was observed: RPVP increased stress concentrations within the cement itself and at the T12 inferior endplate. Critically, lateral bending induced the greatest instability in both techniques, highlighting the necessity for postoperative movement restrictions in this plane.

**Conclusion:**

RPVP significantly reduces cement migration risk during Kümmell's disease treatment through optimized cement distribution. This biomechanical optimization establishes RPVP as a viable alternative to CPVP, enhancing surgical precision and stability while expanding clinical options for vertebral augmentation.

## 1 Introduction

Kümmell's disease, formally classified as delayed post-traumatic vertebral osteonecrosis, represents an uncommon complication of OVCF characterized by progressive collapse following initial injury healing. This condition manifests after varied latency periods—ranging from weeks to years—following an initial vertebral fracture event, attributed to avascular necrosis at the fracture site (1–3). During the subacute phase, patients often present with insidious symptom onset characterized by progressive manifestations: persistent mechanical low back pain, worsening kyphotic deformity, radicular symptoms (lower extremity pain/numbness), neurogenic bladder/bowel dysfunction, and potential motor deficits in advanced stages (4, 5). The diagnostic imaging triad includes vertebral body collapse with loss of anterior/posterior height ratio, IVC on spine radiographs, and “Double-line sign” on T2-weighted MRI (5–7). Amid accelerated global demographic aging, Kümmell's disease affects 7%–37% of OVCF patients, representing an established late complication in approximately one-third of this population (8, 9). Consequently, Kümmell's disease substantially compromises functional independence in elderly patients through progressive vertebral collapse and neurological sequelae, leading to significant deterioration in quality-of-life indices including mobility, pain control, and daily living activities.

For patients with Stage I–II Kümmell's disease without significant neurological compromise, PVP has become a primary minimally invasive treatment option aimed at stabilizing the fractured vertebra, alleviating pain, and preventing further collapse (10–13). However, CPVP has inherent limitations. When bone cement is injected into the IVC, it is difficult to fully interlock and fuse with the underlying cancellous bone, and it tends to form clumps, leading to stress concentration and postoperative loosening of the bone cement. The incidence rate is as high as 25%, and some patients even require revision surgery (14).

In recent years, with the widespread application of orthopedic surgical navigation robots, a new technology-robot-navigated targeted cross-puncture percutaneous vertebroplasty (RPVP) has emerged. Through real-time visual guidance, this technology enables precise puncture. It can not only completely fill the bone cement into the IVC but also further penetrate into the healthy cancellous bone area below the fissure, forming a “cross-fissure cement anchoring” biomechanical anchoring structure. This “cross—fissure anchoring” mode helps to disperse the load, reduce the risk of bone cement displacement, and enhance the overall stability of the vertebral body. In addition, RPVP can be completed under local anesthesia, reducing repeated fluoroscopy exposure and improving the safety and efficiency of the surgery. Although clinical studies have shown that RPVP has good curative effects, there is currently a lack of systematic biomechanical analysis to clarify its mechanism of action. Therefore, this study aims to use the finite element analysis method to compare the biomechanical characteristics of RPVP and CPVP in the treatment of OVCF complicated with Kümmell's disease. The focus is on evaluating the impact of the intra-vertebral anchoring structure on the stability of bone cement, the stress distribution of the vertebral body, and the load of adjacent segments, so as to provide a mechanistic validation for the clinical application of this technology.

## 2 Materials and methods

### 2.1 Data scanning and modeling

A 69-year-old male with L1 Kümmell's disease (symptom duration: 3 months) was prospectively enrolled from our spine surgery department in 2024. Upon admission, comprehensive baseline screening encompassed physical examination, radiographic assessment (x-ray/CT), and laboratory tests to exclude spinal deformities, neoplasms, and infectious processes. A 64-slice helical CT scan of the T12–L2 segment provided DICOM data for patient-specific finite element model reconstruction. Written informed consent was obtained after full explanation of experimental protocols prior to study initiation.

### 2.2 Establishment of L1 Kümmell disease model

**Figure 1:**
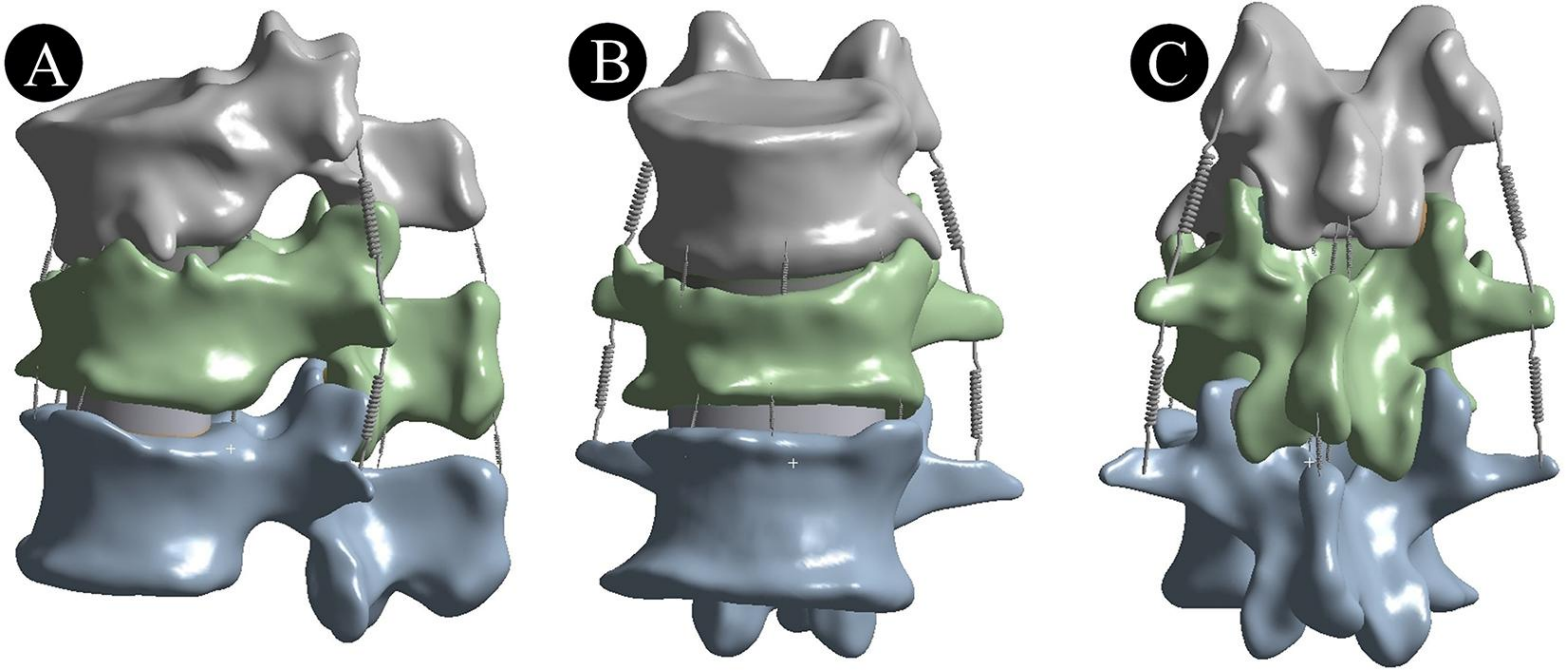
T12–L2 vertebral model including the anterior longitudinal ligament, posterior longitudinal ligament, ligamentum flavum, articular capsule ligament, intertransverse ligament, interspinous ligament, and supraspinous ligament. **(A)** lateral view; **(B)** front view; **(C)** rear view.

Thin-slice CT scanning was performed using a 64-slice spiral CT scanner (GE Company), covering the range from T12–L2. The scanning parameters were set as follows: 140 kv, 200 mA, slice thickness of 0.625 mm, and no interval (15, 16). DICOM data were imported into Mimics software (version 21.0, Materialise company, Leuven, Belgium) for 3D reconstruction. Using Hounsfield unit (HU) threshold-based segmentation, a T12–L2 finite element model was generated through mask editing operations (region growing/erasing) and exported in STL format. Geomagic Wrap 2021 software (Geomagic, USA) processing entailed sequential mesh optimization (remeshing/spike removal/surface smoothing), anatomical facet refinement via digital sculpting, and NURBS surface reconstruction following precise contour modeling. Cortical and cancellous bone regions were segmented using a global offset (−1 mm), generating STL-format models of T12–L2 vertebral structures. Anatomically accurate surface models were reconstructed in SolidWorks 2020 software (Dassault Systemes Company, USA) based on T12–L2 morphology. Vertebral endplates (T12 inferior, L1 superior/inferior, L2 superior) and intervertebral discs were modeled, with Boolean operations creating nucleus pulposus (43% disc volume) and annulus fibrosus compartments (17). An IVC was established by resecting the anterior two-thirds of L1. Ligamentous structures—including anterior longitudinal ligament (ALL), posterior longitudinal ligament (PLL), ligamentum flavum (LF), capsular ligament (CL), intertransverse ligament (ITL), interspinous ligaments (ISL), and supraspinous ligament (SSL)—were simulated using LINK180 elements in ANSYS Workbench 20 (USA). Finally, the above-generated model was assembled into a complete osteoporotic vertebral body model of the T12–L2 segment (18). As shown in Figure 1.

### 2.3 Bone cement distribution modeling

**Figure 2:**
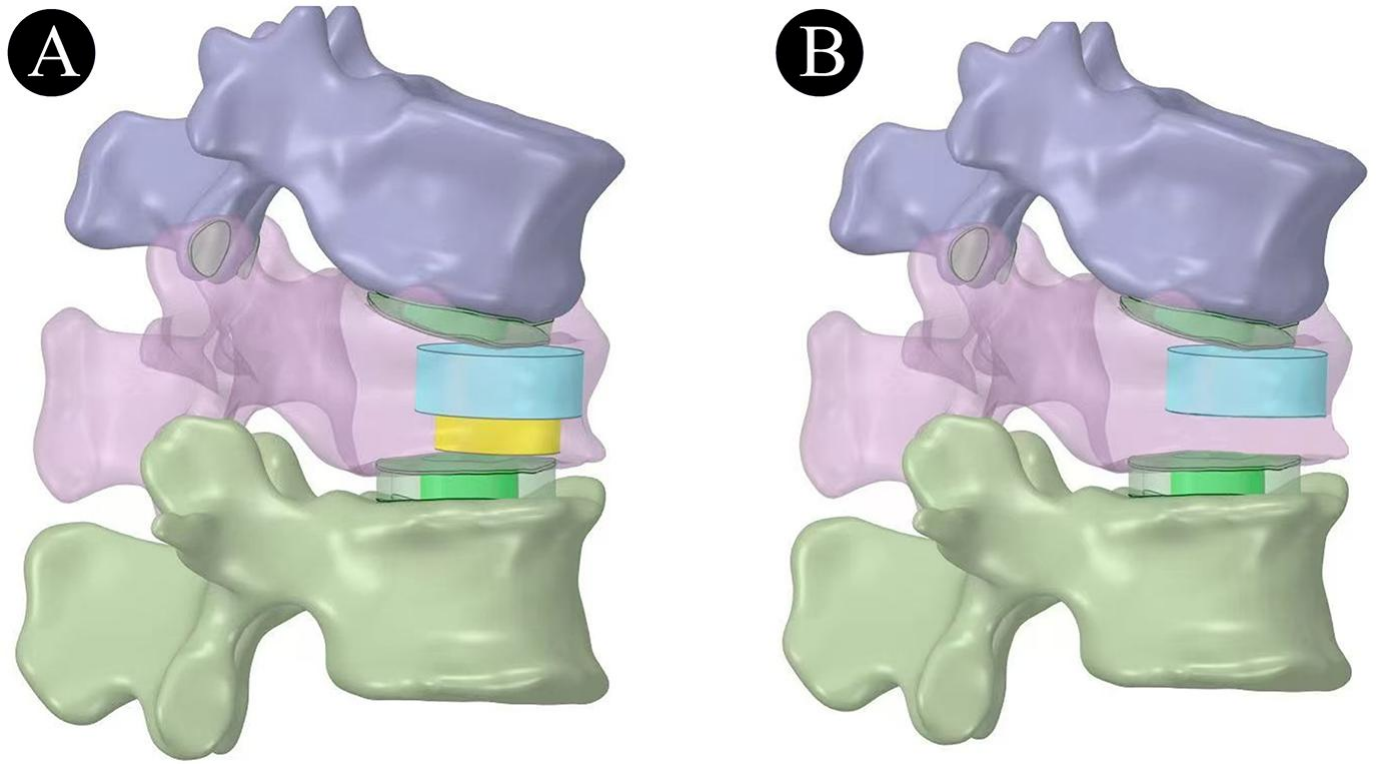
Bone cement distribution model (**A**: RPVP; **B**: CPVP). Blue indicates that the bone cement completely fills the IVC, and yellow indicates the bone cement in the cancellous bone below the IVC.

One or two simulated bone cement cylinders were implanted into the fractured L1 vertebral body to simulate cement distribution patterns. For CPVP, a single 4 mL cement cylinder was positioned with its centroid at the anterior third of the vertebral body, completely occupying the IVC. The RPVP incorporated an additional 1.5 mL cement cylinder injected into subjacent cancellous bone—also centered at the anterior third—establishing mechanical anchoring within the vertebral body. As shown in Figure 2.

### 2.4 Handling of model meshing

Both model datasets were imported into HyperMesh software(Altair Engineering) for body meshing. Given the complex geometry of vertebral structures, tetrahedral Solid187 elements were selected to accommodate anatomical irregularities. The mesh size of all the cells was set to 1 mm, and the number of nodes and cells generated by the two T12–L2 segmental finite element models are shown in Table 1.

**Table 1:** Number of nodes and cells generated for the spine finite element model.

Groups	Node number	Cell number
RPVP	1,90,668	44,110
CPVP	1,91,235	44,742

### 2.5 Material parameters

Referring to previous literature (19–25), the material property parameters for cortical bone, cancellous bone, injured vertebral cortical bone, injured vertebral cancellous bone, upper and lower endplates, nucleus pulposus, annulus fibrosus, bone cement, and related ligament materials in the model are assigned as shown in Table 2.

**Table 2:** Material parameters of the finite element model.

Materials	Modulus of elasticity (MPa)	Poisson ratio	Stiffness (*N*·mm)
Normal cortical bone	12,000	0.3	
Osteoporotic cortical bone	8,040	0.3	
Normal cancellous bone	132	0.2	
Osteoporotic cancellous bone	34	0.2	
Normal end plate	1,000	0.4	
Osteoporotic endplate	670	0.4	
Nucleus pulposus	1.0	0.49	
Fibrous ring matrix	4.2	0.45	
Anterior longitudinal ligament			8.74
Posterior longitudinal ligament			5.82
Yellow ligament			15.75
Joint capsule ligament			10.85
Transverse ligament			15.38
Intervertebral ligament			0.19
Superior spinal ligament			2.39
PMMA	3,000	0.4	

### 2.6 Boundary conditions and load settings

Finite element meshing and material property assignment were performed using HyperMesh software. The T12–L2 vertebral model was exported in CDB format and imported into ANSYS Mechanical (2020 R1, ANSYS Inc.) for biomechanical simulation under standing posture conditions, with applied loads and boundary constraints defined per established protocols.

The interfaces between cortical and cancellous bone, nucleus pulposus and annulus fibrosus, bone cement and cancellous bone, bone cement and itself, vertebral body and disc components, intervertebral disc and endplate, as well as cortical bone and endplate, were defined as bonded contacts. The relationship between the small joints and the posterior structures is set as a non-separable contact relationship, with a friction coefficient of 0.1. The lower surface of the L2 vertebral body is set as fully constrained to define the boundary conditions; a vertical load of 500 N is applied to the upper surface of the T12 vertebral body to simulate the standing posture. Additionally, a torque of 7.5 Nm is applied in the +X direction to simulate flexion, and in the −X direction to simulate extension; torque applied in the +Y and −Y directions simulates left and right bending, respectively; torque applied in the +Z and −Z directions simulates left and right rotation, respectively.

### 2.7 Observation indicators

Under varying loading conditions, the following biomechanical parameters were quantified: peak Von Mises stress at the T12 inferior endplate; maximum Von Mises stress and relative displacement of bone cement within the L1 vertebral body; pk Von Mises stress in the L1 vertebral body; and maximum relative displacement of the T12–L2 functional spinal unit.

### 2.8 Key observation indicators

Under different loads, the maximum Von Mises stress was observed in the L1 vertebrae of the two models mentioned above and in the injected bone cement. Von Mises stress is obtained based on the principal stresses in three directions (X, Y, and Z directions), and the calculation method is as follows:

$$\sigma =\sqrt{[(\sigma 1-\sigma 2)2+(\sigma 2-\sigma 3)2+(\sigma 3-\sigma 1)2]/2.}$$

Where *σ* is the Von Mises stress, and *σ* 1, *σ* 2, and *σ* 3 are the principal stresses in the X, Y, and Z directions of the model, respectively.

## 3 Results

### 3.1 Validation of the finite element analysis model

**Figure 3:**
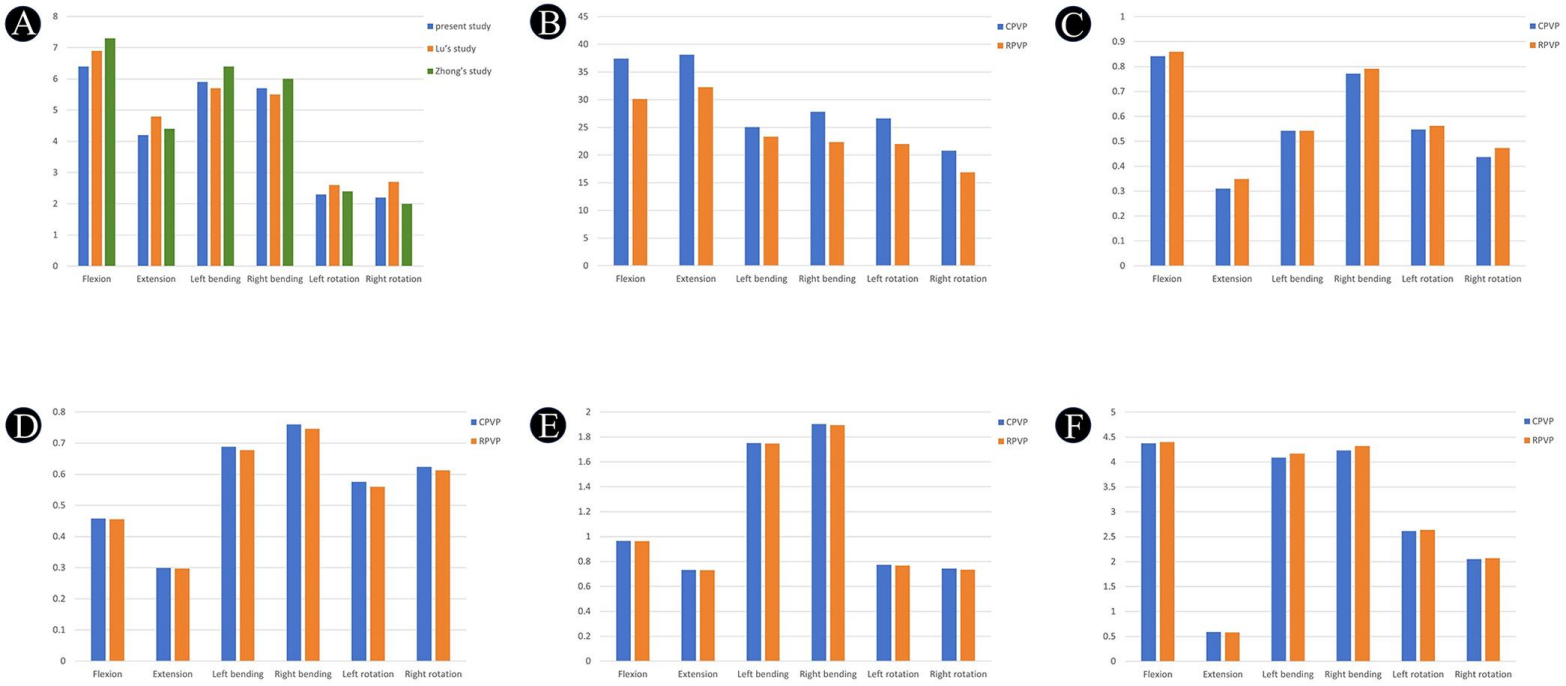
**(A)** Comparison of the range of motion (ROM) of T12–L2 in the model produced in this study with previously reported data. **(B)** Histogram of the maximum Von Mises stresses in the L1 vertebrae of the models. **(C)** Histogram of the maximum Von Mises stress in bone cement. **(D)** Histogram of the maximum relative displacement distance in bone cement. **(E)** Relative displacement distance cloud of the T12–L2 functional spinal unit. **(F)** Histogram of the maximum Von Mises stress on the T12 inferior endplate.

The L1 Kümmell's disease finite element model was validated under six loading directions (flexion, extension, left/right lateral bending, and left/right axial rotation). The resulting T12–L2 range of motion (ROM) data demonstrated close agreement with established biomechanical studies, confirming model validity for simulating post-PVP biomechanics (26, 27). As shown in Figure 3.

### 3.2 Peak Von Mises stress distribution in L1 vertebral body

**Figure 4:**
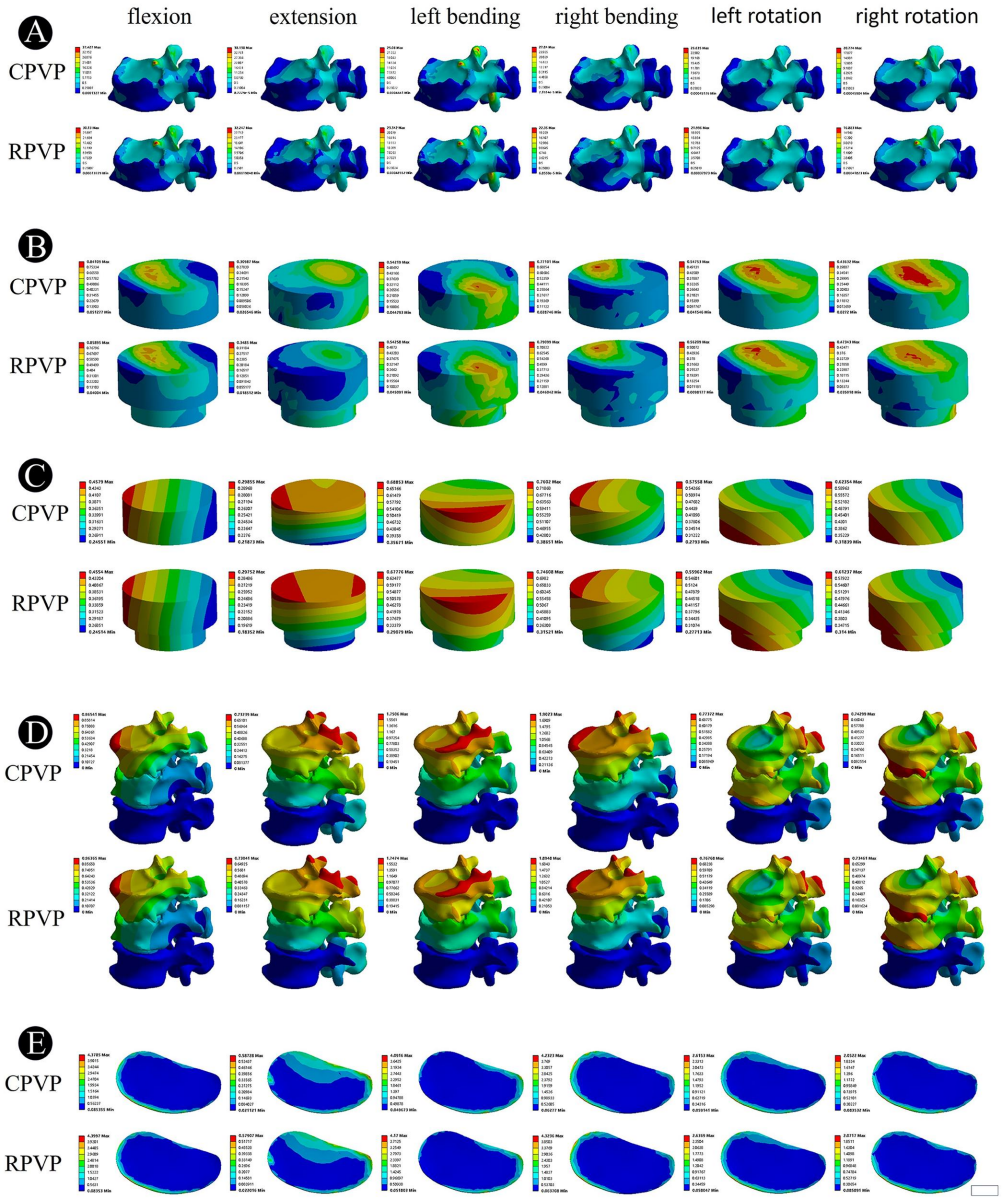
**(A)** Stress clouds of L1 vertebrae in flexion, extension, left/right lateral bending, and left/right axial rotation in two sets of models. **(B)** Stress clouds of bone cement in flexion, extension, left/right lateral bending, and left/right axial rotation in two sets of models. **(C)** Displacement clouds of bone cement in flexion, extension, left/right lateral bending, and left/right axial rotation in two sets of models. **(D)** Displacement clouds of the T12-L2 functional spinal unit in flexion, extension, left/right lateral bending, and left/right axial rotation in two sets of models. **(E)** Stress clouds of the T12 inferior endplate in flexion, extension, left/right lateral bending, and left/right axial rotation in two sets of models.

Maximum Von Mises stress contour plots for the L1 vertebrae in the two groups are shown in the Figure 4. The computational results and statistical histograms after applying the same load are shown in the Figure 3 and Table 3. Based on these results, the stress distributions in the two groups differ significantly under flexion, extension, lateral flexion, and rotation conditions. In contrast, the maximum Von Mises stress values of the L1 vertebra in the RPVP are lower than those in the CPVP under flexion, extension, lateral flexion, and axial rotation conditions, indicating better relative stability of the L1 vertebra. The stress values are as follows: flexion, 30.130 MPa; extension, 32.247 MPa; left lateral flexion, 23.342 MPa; right lateral flexion, 22.350 MPa; left axial rotation, 21.996 MPa; and right axial rotation, 16.883 MPa.

**Table 3:** **(A)** Maximum Von mises stresses in the vertebral body of both models after percutaneous vertebroplasty for L1 fracture. **(B)** Maximum Von Mises stress of bone cement within the vertebral body following percutaneous vertebroplasty for L1 fractures. **(C)** Maximum relative displacement of bone cement in the vertebral body following percutaneous vertebroplasty for L1 fractures. **(D)** Maximum relative displacement of the T12–L2 functional spinal unit following percutaneous vertebroplasty for L1 fractures. **(E)** Maximum Von Mises stress at the inferior endplate of T12 following percutaneous vertebroplasty for L1 fractures.

	Groups	Flexion	Extension	Left bending	Right bending	Left rotation	Right rotation
A	CPVP	37.427	38.138	25.060	27.840	26.635	20.774
	RPVP	30.130	32.247	23.342	22.350	21.996	16.883
B	CPVP	0.841	0.310	0.542	0.771	0.548	0.436
	RPVP	0.859	0.349	0.543	0.791	0.562	0.473
C	CPVP	0.4579	0.2986	0.6885	0.7602	0.5756	0.6235
	RPVP	0.4554	0.2975	0.6778	0.7461	0.5596	0.6124
D	CPVP	0.9654	0.7324	1.7506	1.9023	0.7737	0.7430
	RPVP	0.9637	0.7304	1.7474	1.8948	0.7679	0.7346
E	CPVP	4.3785	0.5873	4.0916	4.2323	2.6153	2.0522
	RPVP	4.3997	0.5791	4.1700	4.3236	2.6369	2.0717

### 3.3 Von Mises stress and displacement distance of bone cement in the L1 vertebral body

The maximum Von Mises stress distribution maps of bone cement in the L1 vertebrae of the two groups are shown in the Figure 4. The computational results and statistical histograms after applying the same load are shown in the Figure 3 and Table 3. Based on these results, there are significant differences in stress distribution between the two groups under conditions of flexion, extension, lateral flexion, and axial rotation. In contrast, the maximum Von Mises stress values of bone cement in the L1 vertebrae of the RPVP were higher than those of the CPVP during flexion, extension, left/right lateral bending, and left/right axial rotation, indicating that bone cement in the RPVP bore more stress. The stress values are as follows: flexion, 0.8590 MPa; extension, 0.3485 MPa; left lateral flexion, 0.5426 MPa; right lateral flexion, 0.7910 MPa; left axial rotation, 0.5621 MPa; and right axial rotation, 0.4734 MPa.

After loading the same load, the maximum relative displacement distance of the L1 vertebral body in the RPVP was smaller than that in the CPVP under flexion, extension, lateral flexion, and rotation conditions, as shown in the Figures 3, 4 and Table 3, indicating that the RPVP had better bone cement stability and a lower risk of bone cement loosening or displacement under various conditions. The relative displacement distances of bone cement between the RPVP and the CPVP were as follows: flexion, −0.0025 mm; extension, −0.0011 mm; left lateral flexion, −0.0107 mm; right lateral flexion, −0.0141 mm; left axial rotation, 0.0160 mm; and right axial rotation, −0.0111 mm.

### 3.4 Total maximum relative displacement distance between T12 and L2

The displacement distances between the two groups under flexion, extension, lateral bending, and axial rotation showed no significant differences. As shown in Figures 3, 4, and Table 3. In contrast, the overall maximum relative displacement distances in the RPVP were slightly smaller than those in the CPVP during flexion, extension, lateral flexion, and axial rotation, indicating better overall stability in the RPVP. The displacement distances were as follows: flexion, −0.0017 mm; extension, −0.0020 mm; left lateral flexion, −0.002 mm; right lateral flexion, −0.0075 mm; left axial rotation, 0.0058 mm; and right axial rotation, −0.0084 mm.

### 3.5 Maximum Von Mises stress at the bottom plate under T12

The maximum Von Mises stress contour plots of the T12 endplate in the two groups are shown in the Figure 4. The computational results and statistical histograms after applying the same load are shown in the Figure 3 and Table 3. Based on these results, the stress distributions under flexion, extension, lateral flexion, and axial rotation conditions are similar between the two groups. In contrast, the maximum Von Mises stress value at the endplate of T12 in the RPVP was slightly higher than that in the control group, indicating that the Von Mises stress value on the T12 vertebral body in the RPVP was slightly higher than that in the CPVP. The stress values are as follows: flexion, 4.3997 MPa; extension, 0.5791 MPa; left lateral flexion, 4.1700 MPa; right lateral flexion, 4.3236 MPa; left axial rotation, 2.6369 MPa; and right axial rotation, 2.0717 MPa.

## 4 Discussion

Kümmell disease was first described by German surgeon Hermann Kümmell in 1895. In this condition, the blood supply to the anterior third of the vertebral body primarily originates from a single terminal artery. In patients with osteoporosis, even minor trauma can easily disrupt blood flow to this region, leading to non-union of fractures in the anterior third of the vertebral body. This, in turn, causes local ischemia and bone necrosis. Kümmell disease is also known as delayed ischemic osteonecrosis, ultimately resulting in vertebral compression collapse (28, 29). Initially, it was believed that conservative treatment could be prioritized when Kümmell disease presented without significant neurological symptoms. However, given that osteonecrosis leads to nonunion of the vertebral body, which cannot heal spontaneously (forming an IVC), conservative treatment typically fails to produce satisfactory outcomes. Additionally, vertebral compression collapse and kyphosis pose a high risk of neurological dysfunction, making surgical intervention necessary (30). With the rapid advancement of minimally invasive techniques, for patients with Stage I and II Kümmell disease who have not yet developed significant neurological symptoms, the use of bone cement to fully fill the IVC and restore vertebral strength represents the least invasive and safest treatment option for Kümmell disease. Therefore, PVP is one of the primary minimally invasive surgical treatment modalities (31). The aim is to eliminate IVC and restore the biomechanical properties of the vertebrae, alleviate pain, prevent the progression of kyphotic deformity, enable a swift return to normal daily activities, and avoid prolonged bed rest (32).

Research indicates that IVC is an independent risk factor for re-fracture of the injured vertebra in OVCF patients following PVA surgery, and it is also the primary determinant of bone cement leakage risk (33, 34). Therefore, with the widespread application of PVP technology, issues such as bone cement leakage, postoperative bone cement loosening, and further compression collapse of the vertebral body have emerged, and in severe cases, catastrophic complications such as bone cement displacement may occur (34, 35). In cases of bone cement displacement, surgeons typically need to perform revision surgery on the injured vertebrae via anterior, posterior, or combined approaches to remove the bone cement and restore spinal stability, thereby reducing back pain in patients. This can cause greater trauma for elderly patients, and those with multiple underlying conditions may not tolerate surgery. Additionally, bed rest can further accelerate osteoporosis in elderly patients, severely impacting their quality of life in later years. CPVP uses bone cement to fill the IVC until the fissure is completely filled. Reactive sclerotic bone forms around the necrotic vertebra, and the bone cement cannot diffuse well into the cancellous bone, forming clumps that do not anchor well (34). Furthermore, in patients with osteoporotic vertebral compression fractures, the trabecular bone beneath the fracture gap is fragile and sparse, failing to provide adequate support for the bone cement. The high stress from the bone cement can lead to further compression and collapse of the injured vertebra, significantly impairing the long-term efficacy of the surgery and patient satisfaction with PVP. Currently, to address cement loosening or displacement, some scholars have adopted PVP combined with pedicle fixation, i.e., injecting cement into the IVC followed by injection into the pedicle to create a “tail sign”, which can enhance cement stability to some extent (36, 37). However, this technique heavily relies on the surgeon's experience and skill. If the pedicle is perforated during surgery or is already damaged or fractured, the bone cement is highly likely to leak into the spinal canal. In mild cases, this may cause artificial spinal stenosis and stimulate spinal nerves; in severe cases, open surgery may be required to remove the bone cement and perform spinal decompression surgery. Additionally, since the anchored bone cement forms a horizontal arm with the bone cement in the IVC, significant stress is distributed across the horizontal arm of the bone cement, posing a risk of fracture. The effect of counteracting the sinking of the bone cement mass is relatively limited. Furthermore, some scholars have employed unilateral or bilateral pedicle screws combined with PVP for Kümmell disease treatment (38). This technique requires general anesthesia and involves the dissection of muscles and fascia during surgery, resulting in significant trauma. Given that elderly patients often have multiple underlying conditions, this approach inevitably increases various risks for patients and is not suitable for those who cannot tolerate surgery.

With the widespread adoption of minimally invasive concepts and the extensive application of orthopedic surgical navigation robots, we are not only able to precisely access the IVC under visualization, but also, when necessary, provide gelatin sponge filling at the site of cortical bone fractures to reduce the occurrence of bone cement leakage. Additionally, we can precisely access the cancellous bone below the IVC, achieving bilateral pedicle cross-puncture to secure the vertebral body, thereby achieving the “top-to-bottom” effect. We refer to this as the RPVP technique, which can be performed under local anesthesia and does not require repeated fluoroscopy under the assistance of orthopedic navigation robots, offering greater benefits to both patients and medical staff. The RPVP technique involves completely sealing the IVC and injecting bone cement into the cancellous bone below the fissure cavity, providing upward support for the bone cement within the IVC. This slows down further compression and collapse of the vertebral body, indirectly enhancing bone cement anchorage and vertebral stability. However, there is a lack of biomechanical studies elucidating the underlying mechanisms.

This study utilized finite element analysis to investigate the biomechanical effects of vertebral body anchoring on injured vertebrae, focusing on the biomechanical characteristics of spinal surgery in daily life. The results of this study showed that the maximum Von Mises stress value of the L1 vertebra in the RPVP was lower than that in the CPVP, indicating that the postoperative stability of the surgical vertebra was better in the RPVP. Due to the block-like distribution of bone cement within the IVC, stress concentration occurs, and since the RPVP received a greater volume of bone cement, the bone cement in the RPVP endures greater stress than that in the CPVP. Under identical loading conditions, the bone cement in the RPVP can absorb part of the stress, thereby enhancing the stability of the injured vertebra post-surgery. The results of this study show that under various load conditions, the maximum relative displacement distance of bone cement in the RPVP was smaller than that in the CPVP, suggesting that the bone cement in the RPVP had better stability, a lower risk of loosening or displacement, and a better prognosis. Additionally, under the same load conditions, the maximum displacement distance of the T12–L2 segment in the RPVP was slightly smaller than that in the CPVP, indicating better overall stability of the vertebral bodies. However, we observed a slightly higher maximum Von Mises stress at the T12 inferior endplate in the RPVP group, indicating a potentially increased biomechanical load on the adjacent cranial segment compared to CPVP. Studies have shown that IVC itself is one of the risk factors for adjacent vertebral fractures, and simultaneously, bone cement leakage into the intervertebral disc is also one of the important risk factors for adjacent vertebral fractures. The results of this study also showed that under the hyperextension condition, the maximum Von Mises stress at the T12 endplate was significantly lower than under other conditions. Since the bone cement mass was located in the anterior 2/3 of the L1 vertebral body, this result indirectly confirmed that the stress concentration of the bone cement mass has a greater impact on adjacent vertebrae. Additionally, we found that, regardless of whether it was the maximum relative displacement of bone cement within the injured vertebra or the overall maximum relative displacement between T12 and L2, the relative displacement was the largest and stability was the poorest under left and right lateral bending conditions compared to other conditions. Therefore, for patients with Kummell's disease, post-operative restrictions on lateral bending activities should be given greater attention.

This study still has limitations: first, only one patient was selected, and finite element analysis was used, so the data results lack universality and may be biased due to individual differences. Second, the simulated working conditions were only for everyday standing positions, and the results cannot be predicted if a greater force is suddenly applied. The next step in the research should be to utilize high-resolution imaging and advanced segmentation techniques to perform more detailed, patient-specific reconstructions of IVC morphology. Meanwhile, finite element analysis should be pursued using multiple actual IVCs from patients with Kümmell's disease.

## 5 Conclusion

Under the guidance of an orthopedic navigation robot, targeted cross-puncture can effectively prevent cement loosening and displacement during the treatment of Kümmell disease. This modified surgical procedure offers a reliable treatment option for PVP in the management of Kümmell disease, providing patients with an additional clinical choice for treatment.

## Data Availability

The original contributions presented in the study are included in the article/Supplementary Material, further inquiries can be directed to the corresponding author.
